# Complement Therapeutics in the Multi-Organ Donor: Do or Don't?

**DOI:** 10.3389/fimmu.2019.00329

**Published:** 2019-02-27

**Authors:** Judith E. van Zanden, Neeltina M. Jager, Mohamed R. Daha, Michiel E. Erasmus, Henri G. D. Leuvenink, Marc A. Seelen

**Affiliations:** ^1^Department of Surgery, University Medical Center Groningen, Groningen, Netherlands; ^2^Department of Nephrology, Leiden University Medical Center, Leiden, Netherlands; ^3^Division of Nephrology, Department of Internal Medicine, University Medical Center Groningen, Groningen, Netherlands; ^4^Department of Thoracic Surgery, University Medical Center Groningen, Groningen, Netherlands

**Keywords:** complement system, complement therapeutics, donor management, organ donor, deceased after brain death, deceased after circulatory death

## Abstract

Over the last decade, striking progress has been made in the field of organ transplantation, such as better surgical expertise and preservation techniques. Therefore, organ transplantation is nowadays considered a successful treatment in end-stage diseases of various organs, e.g. the kidney, liver, intestine, heart, and lungs. However, there are still barriers which prevent a lifelong survival of the donor graft in the recipient. Activation of the immune system is an important limiting factor in the transplantation process. As part of this pro-inflammatory environment, the complement system is triggered. Complement activation plays a key role in the transplantation process, as highlighted by the amount of studies in ischemia-reperfusion injury (IRI) and rejection. However, new insight have shown that complement is not only activated in the later stages of transplantation, but already commences in the donor. In deceased donors, complement activation is associated with deteriorated quality of deceased donor organs. Of importance, since most donor organs are derived from either brain-dead donors or deceased after circulatory death donors. The exact mechanisms and the role of the complement system in the pathophysiology of the deceased donor have been underexposed. This review provides an overview of the current knowledge on complement activation in the (multi-)organ donor. Targeting the complement system might be a promising therapeutic strategy to improve the quality of various donor organs. Therefore, we will discuss the complement therapeutics that already have been tested in the donor. Finally, we question whether complement therapeutics should be translated to the clinics and if all organs share the same potential complement targets, considering the physiological differences of each organ.

## Introduction

### Donor Condition

**Figure 1:**
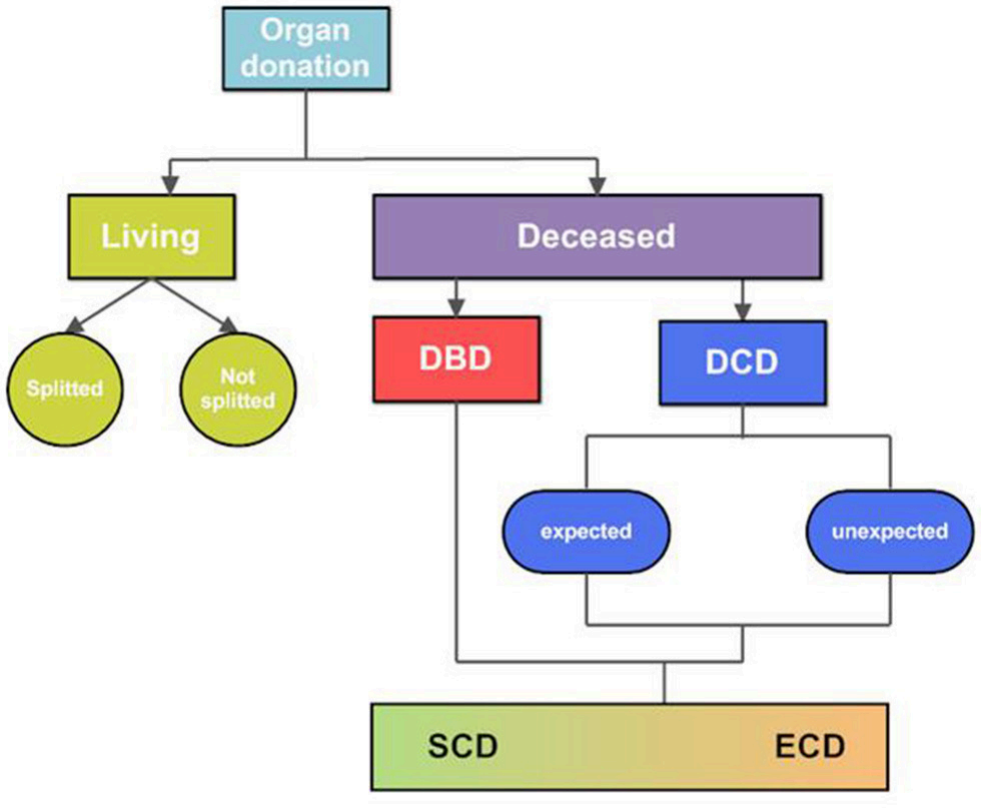
Overview of donor types. Organs are retrieved from living and deceased organ donors. Organs from living donors can be donated partially, so-called “splitted” organ donation, or as a whole organ. The rest of the organs are retrieved from deceased donors, either from brain death (DBD) donors or circulatory death (DCD) donors. The DBD donor includes brain death with an intact circulation and preserved respiration. DCD donation refers to a donor with a cardiac arrest or loss of cardiac function, occurred before procurement of the organs. DCD donation can be divided in both expected and unexpected donation. Expected DCD donation refers to the procurement of organs after a planned withdrawal of life-sustaining treatments. Unexpected DCD donation refers to a donor with unexpected cardiac arrest, from which this donor could not be resuscitated. The quality of organs retrieved from deceased donors are variable, therefore deceased organs are classified into two groups: standard-criteria donors or extended-criteria donors (ECD). This subdivision is introduced to reflect the quality of the organ, of which ECD include potential donor organs that do not match standard donor criteria. DBD, donation after brain death; DCD, donation after circulatory death; SCD, standard criteria donor; ECD, extended criteria donor.

**Figure 2:**
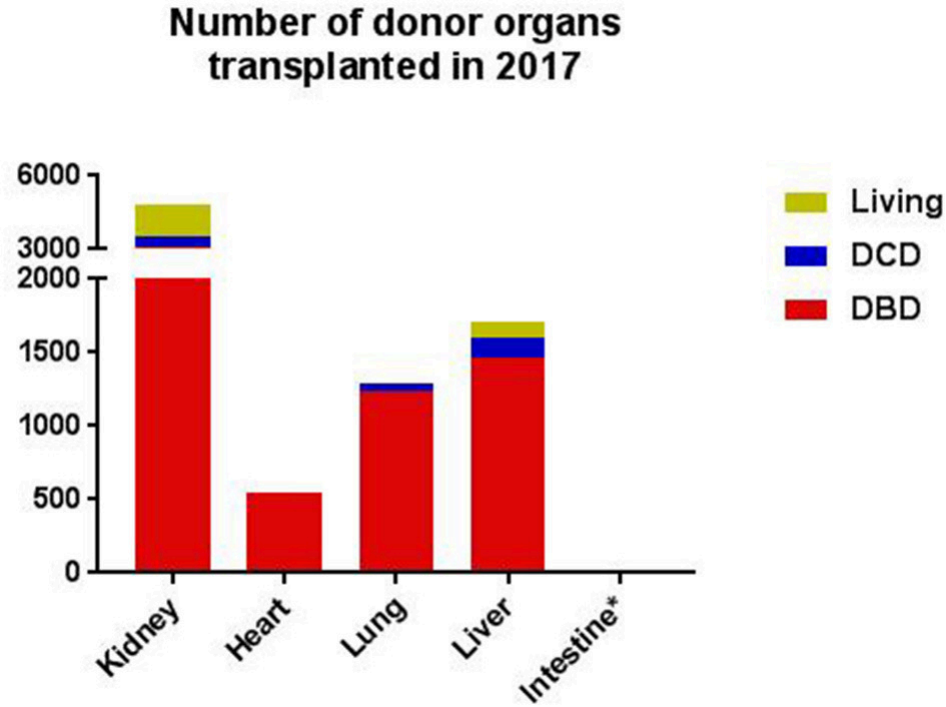
The number of donor organs transplanted in 2017, as documented by Eurotransplant, per type of donor, per organ (4, 5). *In 2017, the number of intestines donated was six. DBD, donation after brain death; DCD, donation after cardiac death. The data was consulted on the 25th of October 2018, permission for publication was obtained.

Organ transplantation is the gold standard treatment for end-stage diseases in various organs, including the kidney, liver, intestine, heart, and lungs (1). The field of organ transplantation has made enormous progress over the last decades. From immunosuppression and tissue matching to organ procurement and preservation, all these developments significantly contributed to the progress made in the field of transplantation (2). Nevertheless, donor availability and quality are still important limitations. Organs are mostly retrieved from deceased donors, and to much lesser extent from living donors (Figure 1). Deceased donors include donation after brain death (DBD) and donation after circulatory death (DCD). The DCD donor can be divided into “expected” and “unexpected”. Expected DCD donation takes place after planned withdrawal of life-sustaining ventilator support. In contrast, unexpected death refers to a donor who had an unanticipated cardiac arrest, without successful resuscitation. Due to the seizure of circulation, DCD-derived organs have a variable period of warm ischemia time prior to retrieval (3). Since the past few years, the number of DCD donors is increasing, in particular in Belgium, Spain, The United Kingdom and The Netherlands. However, within Europe, most organs are still retrieved from DBD donors (Figure 2).

Brain death is the irreversible, total loss of brain function. Due to artificial ventilation and cardiovascular support, the circulation remains intact. Therefore, DBD donors do not have a nominable time of warm ischemia. The occurrence of brain death is an important risk factor for organ quality since brain death is associated with a cascade of hemodynamic, hormonal and immunologic events that become operational after brain injury, or/and brain death (6). Intracranial events leading to brain death promote the disruption of the blood-brain barrier integrity and the neurovascular system, which results in vascular leakage, edema, and hemorrhage. With these changes an immediate rise in intracranial pressure occurs, which causes hypoperfusion of the brain and brainstem. Ischemia of the medulla leads to sympathetic hyperactivation which causes a catecholamine storm, and subsequently a rise of the mean arterial pressure and peripheral vasoconstriction. Activation of the sympathetic nervous system activates the parasympathetic nervous system as well, resulting in bradycardia. This physiological nervous system response is also known as the Cushing response. In addition, brain ischemia results into damage of the hypothalamus-pituitary-axis, causing hormonal depletion. As a consequence, plasma levels of all hormones are lower in DBD donors. Depletion of the antidiuretic hormone increases diuresis as well as the risk for hypovolemia in the DBD donor. Last but not least, the immune system responds quickly to brain injury with both a sterile and non-sterile immune reaction. Concerning the DCD donor, only a few experimental studies have investigated the pathophysiological processes following circulatory arrest in the target organs. These studies show that apoptosis is one of the most important pathways of injury in DCD donors, in contrast to the inflammatory pathways in DBD donors. Whole genome microarray analyses performed by Damman et al. support these findings, which observed enriched NOD-like receptor pathways in DCD reperfusion biopsies. These results suggest that tissue hypoxia in deceased organs leads to cell necrosis and the release of damage-associated molecular pattern molecules (DAMPs). In addition, the study demonstrates that the multiple hypoxia-related pathways found in DCD reperfusion biopsies are related with delayed graft function (DGF) (7).

Currently, deceased donors represent the primary source for transplanted organs. However, the increasing demand for organ transplants mandates for expansion of the donor pool. Therefore, alternative strategies are deployed, such as an increase of the number of living donors, extension of the criteria for deceased donation and improvements in donor management and procurement (8). Still, continuing efforts to improve and preserve donor organs are important, for which interventions in the donor seem a promising strategy. During the last decade, several donor pretreatment interventions have been explored in both animal and human studies, like the effect of thyroid hormone treatment and steroid treatment (9–11). However, no consensus has been reached regarding these donor treatment strategies, due to inconsistent results (12). Nevertheless, (pre)treatment of the deceased donor or the separate donor organs provides us a window of opportunity to improve graft function and graft survival. In the search for the optimal target in the donor, the complement system might play an important role.

## The Complement System

When the complement system was discovered more than a century ago, it was described as “heat-labile components in serum, complementing antibodies in eliminating bacteria.” Nowadays, the complement system is known as a part of the innate immune system that consists of over 50 proteins in plasma and on cell surfaces. In brief, the complement system contains three activation pathways: the classical pathway (CP), lectin pathway (LP), and the alternative pathway (AP). The CP was the first pathway discovered and is activated by antigen-antibody complexes. These complexes are recognized by C1q, inducing a cascade via C2 and C4, leading to the production of CP C3 convertase (C4b2b). The same result is achieved when pattern recognition molecules of the LP [mannose-binding lectin (MBL), ficolins or collectin-11] bind to their MBL-associated serine proteases (MASP) to cleave C2 and C4. The AP is spontaneously activated by hydrolysis of C3 to C3(H_2_O). Factor B is recruited and cleaved by factor D to form the AP C3 convertase (C3bBb). These convertases are intrinsically unstable, but their half-life is lengthened by interaction with factor properdin (P), a positive stabilizing regulator of the AP (13). The C3 convertases formed by the different pathways are responsible for a low grade cleavage of C3, thereby forming C3b. C3b mediates opsonization or binds to the C3 convertase to form the C5 convertase. The C5-convertases cleave C5 into C5a and C5b after which the membrane attack complex (MAC) is formed, via attachment of C5b to C6, C7, C8, and C9. The MAC complex induces the formation of lipophilic complexes in cell membranes which finally leads to cell lysis. Furthermore, C5b-9 induces tissue injury via intra-cellular pro-inflammatory signaling pathways (14). Finally, injury can be amplified by the formed split products C3a and C5a, acting as anaphylatoxins that provoke influx and activation of inflammatory cells (15, 16).

### The Complement System in the Organ Donor

Under normal circumstances, the complement system is strictly controlled by complement regulators to prevent the destruction of healthy cells and tissues (15). However, when this fine balance is disturbed, the activated complement system may result in tissue injury. Damman et al. demonstrated the involvement of complement activation in deceased organ donors (17). In both DBD and DCD donors, increased systemic complement levels of C5b-9 were found in plasma, compared to C5b-9 levels of living donors. These higher complement levels were associated with increased local tissue injury in deceased donors compared to the living counterparts. Deceased donors also have a higher incidence of acute rejection in renal allografts (17). Van Werkhoven et al. showed an additional upregulation of C5a in plasma from DBD donors compared to living controls (18). Furthermore, De Vries et al. demonstrated that soluble C5b-9 (sC5b-9) release is detected in both DBD and DCD donors before reperfusion, but not in living donors (19). These higher levels of sC5b-9 in deceased donors are associated with inferior renal allograft function after transplantation (20). In accordance, systemic C4d and Bb levels were significantly higher in the deceased donor than in the living counterpart, with both complement proteins being associated with sC5b-9 levels in the DBD donor. Interestingly, there was no association seen between MBL and sC5b-9, which suggests that both the CP and AP are involved in brain death injury, but not the LP. However, with the recent findings of the potential bypasses in the complement system, the LP might still play a role in complement activation in the deceased donor (21, 22). Not only in kidneys, but also in lungs it has been demonstrated that plasma complement levels correlate with tissue injury after transplantation. Shah et al. measured plasma complement levels in recipients before transplantation and found an association with risk for acute lung injury and a higher incidence of mortality in lung transplant recipients (23). These studies indicate that activation of the complement system already commences in the donor, and that systemic complement activation may lead to local inflammation of the potential donor graft. As a result of inflammation, the (donor) organs are damaged. For DBD donors it is hypothesized that brain death initiates a sterile immune response through the release of endogenous DAMPS. Those danger signals are released by cells under conditions of cellular stress or tissue injury. In DBD donors, these endogenous DAMPs are either actively secreted via stressed immune cells or passively released from dying brain cells or damaged extracellular matrix, which contributes to CP activation. Sterile inflammation can also cause further tissue destruction by the release of excessive amounts of DAMPs via necrotic cells. As a result, an extension of the local inflammation to the systemic circulation occurs, in a similar manner as is seen during microbial invasion. C1q and properdin will bind to these necrotic cells, which triggers the activation of the complement system. In addition, brain death causes an increase in intestinal permeability, that results in the release of pathogen-associated molecular pattern molecules (PAMPs) like LPS. In this way, the complement system is activated via the AP. In contrast, data referring to the pathogenic mechanisms in DCD-induced organ injury are scarce (24, 25). Taken together, despite the mentioned hypotheses of injury mechanisms, large knowledge gaps exist with regards to this topic. The exact role of complement activation and the involved complement components have not been fully elucidated in different donor organs and different donor types, especially in DCD donors. Of importance, since the physiological differences between organs and donor types might lead to various routes of immunological activation and therefore require different therapeutic approaches. The purpose of this review is to provide an overview of the current knowledge on the complement system in the donor (Table 1), the current existing knowledge gaps and future perspectives on this topic. Furthermore, we will answer the question if complement therapeutic should be clinically applied in the multi-organ donor.

**Table 1:** Overview of the complement targets or therapeutics tested in the organ donor per organ, per type of donor.

**Organ**	**Species**	**Donor type**	**KO/treatment**	**Study**	**Graft injury**
Kidney	Rat	DBD	sCR1	(42)	↓
		DBD	C1-INH	(38)	↓
	Non-human primate	ECD	C1-INH	(41)	Unknown
	Human	DBD	C1-INH	NCT: 02435732	Unknown
Liver	None				
Intestine	None				
Heart	Mouse	DBD	C3^−/−^	(79)	↓
		DBD	CR2-Crry	(78)	↓
Lung	Mouse	DBD	C3aRA	(100)	↓

*Inclusion criteria were: (1) All animal and human models, testing (2) complement targets or therapeutics applied in the organ donor or during preservation, with (3) the donor injury mechanism as a target of interest (e.g., DBD, DCD). Excluded are studies in which complement targets or therapeutics were tested in IRI or rejection as mechanisms of injury. ↓ The treatment resulted in less graft injury*.

## Renal Transplantation

### Challenges in Renal Transplantation

The kidney was the first organ successfully transplanted, in 1954 (26). Nowadays, renal transplantation (RTx) is the optimal treatment for patients with end-stage renal disease. Besides experience in years, the number of performed kidney transplants exceeds the number of all other solid organ transplants. In 2017, 4.419 renal transplants were performed in Europe, as registered by the Eurotransplant International Foundation (Figure 2). However, the number of patients waiting for a renal transplant is extensive and still increasing (27). The growing shortage of donor kidneys led to exploration of alternative strategies. First, the number of living donations increased significantly over the years. Living kidney donation is constantly evolving and goes nowadays beyond relatives, like individuals who donate a kidney to an anonymous recipient, so-called altruistic or Samaritan donation. Besides, special programs are developed, such as the Old for Old program or domino transplantation. In the Old for Old program kidneys of donors over 65 years or older are donated to recipients of the same age, without taking tissue-matching characteristics into account (28). A domino transplant occurs when the removed organ from first recipient is transplanted in a second recipient (29). Furthermore, the donor pool consisting of both DBD and DCD donors is expanded by the use of kidneys from extended-criteria donors (ECD), which refers to older donors and donors with comorbidities (3). Compared to standard-criteria donor (SCD) organs, kidneys from ECD are associated with up to a 2-fold increased risk of DGF, acute rejection, and graft loss (30). Kidneys from older donors are generally more immunogenic than kidneys from young donors, which makes immunomodulatory approaches in organs from ECDs an interesting topic for future research (30). With the increased utility of kidneys from ECDs in the clinics, more randomized controlled trials should be performed, with ECD kidneys included (31). Potentially, these ECD kidneys form a subgroup who can benefit from treatment already introduced in the donor.

### The Complement System in Renal Transplantation

Most of the evidence for activation of the complement system in deceased donors is known from studies which focus on the kidney. Early studies performed by Kusaka et al. detected local C3 deposition in kidney isografts from DBD rats 1 h post-transplantation, while no C3 deposition was seen in living donor controls. C3 deposition was located on the endothelial cells and glomeruli of DBD kidneys and could still be detected at day 5 after RTx (32). In accordance, Damman et al. showed higher renal C3 gene expression rates in DBD rats before transplantation than in living donors (33). No additional C3 gene expression was found after RTx, so renal C3 is deposited as a direct result of brain death. These results were confirmed in the human setting, in which significantly more C3 gene expression was found in kidney biopsies taken from DBD donors than in biopsies from living donors. The results on transcriptional level were supported by immunohistochemistry. C3d deposition was seen in renal grafts from human DBD donors, but not in living donors. Again, no additional C3d deposition was found after ischemia and reperfusion (33). The functional importance of local C3 synthesis is demonstrated by multiple studies. Pratt et al. showed in a murine model that renal allografts lacking C3 production survive more than five times as long as renal allografts that produce C3 (34). In addition, Brown et al. demonstrated that expression of C3 alleles by renal cells in the deceased human donor significantly affect graft survival (35). However, the association between the C3 allotypes and graft survival could not be replicated by others. Varagunam et al. detected no significant differences between the C3 alleles on long-term renal allograft survival in patients (36). These results are in accordance with the study of Damman et al. which observed that donor C3F allotypes are not associated with renal allograft outcome after RTx. Only subgroup analysis within the DCD group revealed a protective effect of the donor C3F allotype for primary non-function. These divergent results could possibly be explained by the differences in sample size and post-transplantation follow-up data (37).

The importance of the complement system is underlined by the in-depth analysis of the gene expression differences between human renal allograft biopsies from living and deceased donors. Significant renal overexpression of many complement components were seen in deceased donor kidneys before reperfusion (38). Primarily complement-related genes of the CP were involved, namely C1q, C1s, C1r, C2, and C4. Factor B, an component of the AP, was upregulated in deceased kidneys. Similar results were seen in the whole genome microarray study performed by Damman et al. This study shows enrichment of both the hypoxia and complement coagulation pathways in DBD kidneys. The same pathways were involved in the DCD kidney, but in a later phase of transplantation, namely during ischemia (7). Focusing on the downstream complement components, the C5a-C5aR-axis seems to play an important role. C5a is not only systemically upregulated in the deceased donor, but Van Werkhoven et al. showed an increased renal tubular expression of the C5a receptor 1 (C5aR1) (18). Altogether, these studies suggest that the local immune activation in the deceased renal allograft is important for the outcome after RTx. Thus, targeted therapy interfering with (local) complement activation before organ recovery or during organ storage is an attractive therapeutic approach. However, assessing local immune activation requires invasive techniques. For that reason, using complement deposition as an indicator for organ damage might not be preferred. A recent study by Schröppel et al. investigated the potential for less invasive markers by evaluating C3a and C5a levels in donor urine. They found that donor urinary C5a levels were correlated with DGF after kidney transplantation. However, no correlation was seen between urinary C3a and post-transplant DGF, despite higher levels of C3a in urine from deceased donors than in urine from healthy controls. Whether other complement proteins measured in urine correlate with graft function, potentially serving as biomarkers, has not yet been elucidated (39).

Already a few complement therapeutics were tested in the deceased kidney donor or during kidney preservation. One of the most potent complement inhibitors is C1-esterase-inhibitor (C1-INH). As a serine protease inhibitor, *in-vitro* data demonstrated that C1 inhibitor modulates activation the classical- and lectin pathway (40–42). Pre-clinical studies with C1-INH in the deceased donor showed promising results. Poppelaars et al. tested a high-dose and low-dose C1-INH in a rat model of brain death in which C1-INH was administered 30 min after confirmation of brain death. High-dose C1-INH treatment of the DBD donor resulted in significantly lower renal pro-inflammatory gene expressions and decreased serum levels of IL-6. In addition, C1-INH led to an improved renal function reflected by lower serum creatinine levels, and less renal injury as demonstrated by lower kidney injury molecule-1 gene expression levels (40). C1-INH is currently tested as a treatment strategy in human DBD donors to improve outcome after RTx (NCT02435732). At this moment, this study is in the phase of recruiting patients. In ECD donors C1-INH treatment might be of potential therapeutic use as well, which is currently being investigated by Fernandez et al. in a non-human primate model (43).

Besides C1-INH, more complement therapeutics are already tested in the deceased donor in experimental setting. Soluble complement receptor 1 (sCR1) was given to DBD rats and treatment with sCR1 before and after confirmation of brain death led in both cases to significantly improved renal allograft function. In addition, treatment with sCR1 led to reduced renal gene expression of IL-6, IL-1β, and TGF-β. These results provide proof that complement inhibition in the donor is effective, even after the confirmation of brain death (44).

Next to the use of complement therapeutics in the donor, already a few studies tested the effect of complement therapeutics during renal preservation. Patel et al. were the first, and evaluated the effect of APT070, also known as Mirococept (45). Mirococept is a membrane-localizing complement regulator, which is a derivate from complement receptor 1. Rat donor kidneys were perfused with Mirococept and subsequently subjected to 16 h of cold storage. After 16 h of cold storage, the kidneys were transplanted into syngeneic recipients. APT070 perfused renal grafts had survival rates of 64% compared to a survival rate of 26% in control-treated renal allografts. Currently, Mirococept is tested in a multicenter randomized controlled trial, in which Mirococept is administered *ex vivo* to deceased donor kidneys. The trial, called EMPIRIKAL, is still ongoing and aims to evaluate the efficacy of Mirococept in reducing the incidence of DGF in renal transplants from deceased donors (46).

Furthermore, Lewis et al. demonstrated that pharmacological targeting the C5aR is also of potential benefit. In this study a C5aR antagonist named A8^Δ71−773^ was used, which targets both the C5aR1 and C5aR2 (47). Donor kidneys were flushed and stored for 2 h with UW or UW + C5aR antagonist. Kidneys treated with the C5aR antagonist had significantly improved renal function and increased graft survival compared to untreated kidneys. In addition, the C5aR antagonist prevented renal injury, reflected by lower gene expression levels of TNF-α and macrophage inflammatory protein-2/CXCL2. C5 was also targeted in a recent study, in which a monoclonal antibody against C5 was used (48). Rat donor kidneys were cold stored for 28 h with or without anti-C5. Treatment with anti-C5 significantly increased the survival rate of the renal allografts from 22% to 100% after 21 days. Another C5 complement inhibitor is the recently generated recombinant anti-C5 antibody called Ergidina, which is coupled to a cyclic-arginylglycylaspartic (RGD) acid-peptide. The RGD peptide has the property to migrate to the ischemic endothelial cell. Thus, when bound to anti-C5 it will not only be able to migrate, but also be able to control ischemia tissue injury. In a study of Durigutto et al. rat donor kidneys were procured and cold stored for 24 h. Thereafter, kidneys were *ex vivo* infused with Ergidina, and stored for either 15 min or 30 min. Results showed that Ergidina was bound to the vascular endothelium of the kidney and already reached a plateau after 15 min. Next, the efficacy of Ergidina was evaluated in a rat model of IRI. Rats received Ergidina 45 min before ischemia and were sacrificed at day 1 or day 4. Ergidina preserved renal function, prevented tissue injury at glomerular and tubular level and prevented C9 deposition in the kidneys at both day 1 and day 4 (49).

Besides anti-C5, Yu et al. also evaluated the effect of AP inhibitor TT30. TT30 is a complement receptor 2/factor H fusion protein. *Ex vivo* preservation with TT30 for 28 h significantly improved renal function and renal graft survival compared to control-treated kidneys. The 21-day graft survival rate was 66% in the TT30 treated group compared to the 100% in the anti-C5 treated group (48). These survival rates did not significantly differ, but imply that next to the AP, activation of the CP or LP might play an role in in ischemia-induced injury.

Based on the studies already performed, it can be hypothesized that the renal allograft is already primed for complement activation in the deceased donor. Therapeutics interfering with complement activation before organ recovery would be an attractive therapeutic approach that deserves further investigation. Most importantly, studies using complement therapeutics in order to prevent renal injury seem to be most effective when the therapeutics are specifically delivered to the site of complement activation. Therefore, with the increased availability of new complement therapeutics, it is crucial to unravel the role of complement system in the deceased donor. Especially, it is essential to learn whether complement therapeutics can be administered in the donor before organ retrieval or if treatment after organ procurement is preferred.

## Liver Transplantation

### Challenges in Liver Transplantation

Currently, more than 1.500 liver transplantations (LiTx) are performed in Europe per year (Figure 2). In general, LiTx is considered for patients which suffer from acute liver failure, end-stage liver disease and primary hepatic malignancy. However, after a rapid growth, the annual number of LiTx has stopped increasing over the last 10 years. An important limitation for the stagnant number of LiTx is donor shortage (50). Therefore, alternatives to DBD donation of liver transplants are more frequently implemented. First, the concept of “split liver transplantation” is used, which enables surgeons to transplant one donor liver into two recipients. However, this technique is only feasible with ideal livers, mostly derived from young DBD donors. Second, there is an increase in living-related liver transplantation. Living donation for adults is still associated with major complications and a substantial risk for the living donor (51). Therefore, both splitting and living LiTx have not gained widespread acceptance. Finally, more extended criteria are introduced such as advanced age, steatosis and DCD liver grafts. Controversy exists about DCD liver transplants, since studies that compare graft outcome from DCD donors with standard DBD donors have been variable. Studies performed so far suggest decreased graft survival in the first year following DCD LiTx (52). Although ECD donation reduces the gap between supply and demand, managing the risk for the recipient is a critical factor in ECD donor livers. ECD livers are vulnerable to hypoxia and tissue injury associated with DGF or graft survival. In order to improve the quality of these suboptimal liver grafts, several studies evaluated the effect of intervention during donor management, such as the administration of steroids, dopamine, and hormone replacement (10, 11, 53). Taken these data into account, the current strategies applied in LiTx have mainly been focusing on treatments to stabilize hemodynamic disorders associated with deceased donation. Yet, their effects on liver graft function and survival remain unknown. Therefore, it could be beneficial to consider other molecular pathways involved, including the complement system.

### Complement System in Liver Transplantation

The number of studies that investigated the role of the complement system in deceased donation for LiTx is scarce. Of interest, since the liver is responsible for the biosynthesis of 90% of plasma complement components and soluble complement regulators (54). Other type of cells including immune cells and endothelial cells produce complement components as well, but their contribution to plasma levels appear to be minor compared to hepatocytes. Moreover, several complement receptors are expressed in the liver; e.g., C5aR, CR1, CR3, CR4, and complement receptor immunoglobulins (CRIg). These complement receptors have multiple functions on different cells in the liver, from inducing the acute phase response to the clearance of C3-opsonized immune complexes. Thus, the complement system is involved in multiple liver diseases, such as transplant-induced injury (55).

The role of the complement system in the deceased liver is mainly investigated in the DBD donor. Rebolledo et al. were the first to show complement activation in DBD donor livers. In this study, rats were subjected to brain death for a period of 4 h. Results showed that DBD donor livers had a significant increase in C3 mRNA levels compared to the control group. Next, rats were pretreated with prednisolone 30 min before induction of brain death as a proof of principle. Pretreatment with prednisolone resulted in a reduced pro-inflammatory state, reflected by lower mRNA levels of IL-6, IL-1β, TNF-α, and MCP-1. In contrast, mRNA levels of C3 were upregulated in the prednisolone treated rats (9). In an additional study, Rebolledo et al. administered prednisolone after confirmation of brain death to investigate treatment potential. Again, prednisolone reduced the levels of pro-inflammatory cytokine gene expression, but did not increase nor decrease the C3 expression compared to untreated DBD rats. These divergent C3 expressions after administration prednisolone are not fully understood, but could be explained by the fact that complement is involved in liver regeneration (11).

So far, only one study investigated the effect of complement inhibitors on the donor liver before transplantation. Bergamaschini et al. studied the potential of C1-inhibitor (C1-INH) treatment during preservation. Porcine livers were removed from donors and perfused with University of Wisconsin (UW), with or without addition of C1-INH, and stored statically at 4°C for 8 h. To assess liver function, livers were subsequently reperfused for 2 h with pig blood on an extracorporeal circuit. Results demonstrated less complement activation, reflected by normal levels of complement haemolytic activity and absence of C3 activation production in both plasma and tissue at time of reperfusion. Morphological analysis of the livers showed significantly decreased inflammation as shown by only a mild increase in portal and lobular inflammatory infiltration, compared to necrotic lesions in the untreated group (56). Taken together, C1-INH treatment during preservation seems to protect the liver against cell injury and inflammation during the preservation phase. Therefore, complement therapeutics in the donor, but also the preservation phase, might be beneficial for the liver.

Important to take into account in LiTx is the process of liver regeneration. Liver regeneration is a process in the liver that allows mature hepatocytes to re-enter the cell cycle, proliferate, and eventually replace lost or damaged hepatocytes (57). Liver regeneration is important for both donors and recipients of liver transplants, especially in the case of a failing remnant liver after “splitted” liver donation or transplantation of a small-for-size liver in the recipient (58). So far, no therapy exists for these patients, so there is a significant need for strategies that stimulate the regenerative capacity of livers. The complement system seems to have a key role in the liver regeneration, primarily during the early phases, were the hepatocyte re-enters the cell cycle and proliferates. Recently performed studies demonstrated an important role for the complement effector proteins, C3a and C5a (59, 60). Strey et al. subjected C3- and C5-deficient mice to a 70% partial hepatectomy model. Deficiency of C3 or C5 led to significantly less liver regeneration, as reflected by fatal liver failure. Reconstitution of effector molecules C3a and C5a resulted in hepatocyte proliferation, which indicates that C3 and C5 are key factors in regeneration of hepatocytes. The precise role of complement in the deceased donor still needs to be elucidated, but ischemia-reperfusion injury (IRI) studies showed an important role for both C5a and C5b-9 in the induction of injury (61, 62). These contradictive results with regards to the role of complement in hepatic IRI vs. liver regeneration emphasize the need for a fine balance between complement activation and inhibition. Therefore, it is important to have a good understanding of the two processes and test potential complement inhibitors in both disease models. A study performed by He et al. evaluated the effect of CR2-Crry in a combined mouse model of total IRI and 70% partial hepatectomy. CR2-Crry is a fusion protein that specifically targets the sites of C3 activation. This study shows that CR2-Crry is able to protect against hepatic IRI alone, however a combination of IRI and partial hepatectomy resulted in significant liver damage and a failure to regenerate compared to WT mice. The failure to regenerate is probably a result of the inability to generate sufficient levels of C3a, C5a, and C5b-9, complement effector molecules important for liver regeneration (63). Given these observations, CR2-CD59 might be a potential complement therapeutic. CR2-CD59 is a fusion protein that migrates to sites of complement activation and specifically inhibits the MAC, without the blockage of other complement components. CR2-CD59 was tested by Marshall et al. in the same mouse model of total IRI and 70% partial hepatectomy as described by He et al. The study performed by Marshall et al. showed that livers treated with CR2-CD59 have less injury, and more hepatic regeneration than control-treated mice. CR2-CD59 mice had a 100% 7-day survival rate, whereas in the CR2-Crry treated group only 40% of the mice survived (64). These results imply that in MAC-induced injury, the regenerative response of the liver is impaired. Therefore, these studies highlight the need for a tailor-made approach to protect the liver against ischemia injury and enhance the regenerative capacity. Although only a few studies are performed, they all point towards the involvement of the complement system in the deceased donor liver. The exact role of complement in the deceased donor liver and its consequences on liver transplant viability and survival remains unknown. Therefore, more studies, especially focused on the complement-dependent balance between injury and regeneration in the liver, need to be conducted.

## Intestinal Transplantation

### Challenges in Intestinal Transplantation

Intestinal transplantation (ITx) is the least common form of organ transplantation. The field of ITx is small with only 6 ITx reported in 2017 by Eurotransplant (Figure 2). ITx is indicated for patients with intestinal failure who suffer from life-threatening complications when using parenteral nutrition. Despite the advances, ITx is still a challenging procedure due to multiple factors. First, the intestine is highly immunogenic because it consists of a large amount of lymphoid tissue, including the patches of Peyer and the mesenteric lymph nodes (65). Second, the intestine carries an enormous bacterial load. These characteristics create a fine balance between the maintenance of tolerance to healthy self-tissue and eliminating invading pathogens. Moreover, the intestinal mucosa is extremely vulnerable to injury, especially hypoxia injury, which is negatively associated with graft outcome (66). As a result of the susceptibility for hypoxia, DCD donor intestinal grafts are not yet accepted for ITx (67). This makes DBD donors the sole source for intestinal grafts. Despite major achievements in intestinal grafts retrieved from DBD donors, such as improved immunosuppression, the physiological abnormal state in the DBD donor still significantly compromises the viability of the intestine. Therefore, it is concerned that ITx is underutilized due to complex pathophysiological processes and difficulties to identify markers for intestinal injury (68).

### The Complement System in Intestinal Transplantation

Hardly any studies are performed to unravel the pathophysiological processes in the intestine of a deceased donor, probably since the number of ITx performed per year is low. However, based on the few studies there are, it is likely that the deceased donor state causes significant alterations in the intestine and affects intestinal barrier function. The degree of permeability already changes in response to a low level of pro-inflammatory cytokines, which results in the translocation of intestinal bacteria. The intestine contains microbial LPS, which is thought to be one of the most potent activators of the AP of the complement system. Although studies showed that LPS from different bacterial strains interact in qualitatively different ways with complement, LPS from gram-negative bacteria indeed induce consumption of complement (69). A study performed by Koudstaal et al. confirmed the involvement of LPS in the DBD donor by using a brain death model for rats. Rats subjected to 4 h of brain death had higher serum levels of LPS and LPS-binding protein (LBP), as evidence of endotoxemia. Besides, mRNA gene expression levels of LPB were significantly higher in DBD rats than in living controls. DBD rats had a high inflammatory state, reflected by the strongly elevated levels of IL-6 and MCP-1 (70). The study shows enhanced intestinal permeability in DBD rats which results in a high immunological response. The observation that brain injury can rapidly induce significant damages to the intestine is demonstrated by a study of Hang et al. This study investigated the histopathological alterations of the intestinal mucosa in rats after 3–72 h following brain injury. The intestinal mucosa was already severely damaged after 3 h, reflected by shedding and apoptosis of epithelial cells, mucosal atrophy, and loss of increase in intestinal permeability. The level of plasma endotoxin was positively related to the degree of intestinal permeability. Compared with the control group, serum endotoxin levels were significantly increased at 3, 12, and 24 h with a maximal peak at 72 h. The first peak of endotoxin levels, at 3 h, might be the result of acute gut mucosal damage due to ischemia-induced sympathetic hyperactivation. The second peak of serum endotoxin might be induced by mucosal damage and increased epithelial necrosis, which occurs at 72 h (71). Whether complement is activated in the intestine of both the DBD and DCD donor remains to be elucidated. However, the current findings indicate that protection of the intestine in the multi-organ donor is necessary, since translocation of intestinal bacteria and endotoxin lead to a systemic inflammatory response syndrome and sepsis with subsequent multi-organ failure (72). Further research needs to focus on the exact role of complement activation in the deceased intestinal donor. This could not only create a new window of opportunity for immunosuppressive strategies, but also improve the clinical success of ITx.

## Heart Transplantation

### Challenges in Heart Transplantation

The technique for heart transplantation (HTx) was already developed in 1967. Nevertheless, it took more than a decade before immunosuppressive treatment strategies improved this technique to such an extent, that HTx became the gold standard treatment for end-stage heart diseases (73). In 2017, 548 heart transplants were performed in Europe, as registered by the Eurotransplant International Foundation (Figure 2). Both non-ischemic and ischemic cardiomyopathy are the underlying diagnoses responsible for over 80% of heart transplants (74). The increased need for HTx over the years led to the inevitable gap between donor demand and supply, analogous to other donor organs. Despite the attempt to minimize this gap by the introduction of techniques such as left ventricular assist devices (LVAD), these therapies mainly serve as a short-term, “bridge-to-transplant” solution.

Unfortunately, the majority of potential heart donors is not procured and transplanted, for several reasons. First, most countries are limited to the use of DBD hearts, and do not utilize the DCD donor pool. Anxieties exist concerning warm ischemic injury to the myocardium after circulatory death, together with the inability to assess heart function. However, new techniques are being developed to tackle these issues. In an attempt to limit warm ischemia times, implementation of techniques like *in situ* normothermic regional perfusion for thoracic organs are explored in order to convert from a DCD to a DBD-type procurement (75). In addition, normothermic regional perfusion provides the opportunity to functionally assess the donor heart. Furthermore, implementation of techniques such as *ex situ* heart perfusion are being studied (76). Despite its experimental nature it is suggested that those techniques lead to usage of more donor hearts, with comparable outcomes to the current gold standard of DBD HTx (77). A second reason for low procurement rates of potential donor hearts is the relatively strict cardiac donor selection criteria. Age < 55 years old, appropriate hemodynamics and limited inotropic support are examples of selection criteria that impede suitability (78). Godino et al. showed that hemodynamic dysfunction represented the major cause for unsuitability of heart donors, a complication that occurs frequently in DBD donors (79). In terms of immunology, experimental transplantation models have shown that hearts have differences in rejection patterns compared to abdominal organs such as kidneys and livers, leading to higher rejection rates (73). These organ-specific differences in immunology might contribute to the differences seen in graft-survival rates between organs in human transplantation. Of importance, since the immunologically active state of the organ already commences in the donor. The DBD donor has shown to exacerbate post-transplantation cardiac IRI that reduces allograft survival, in which the complement system might play a key role (80).

### The Complement System in Heart Transplantation

The role of the complement system in the deceased heart donor has only been studied in DBD donation, which raises questions for involvement in DCD donation. An experimental mouse study performed by Atkinson et al. showed increased local complement C3d deposition in the heart after brain death. C3d deposition was primarily seen in the vascular endothelium and surrounding myocytes, in a significantly higher amount than in grafts from sham-operated mice. In addition, the study demonstrated that absence of C3 reduced cardiac damage, reflected by less endothelial swelling and lower serum levels of cardiac troponin I. Also, significantly less leukocytes infiltrated the heart tissue and gene expression of P-selectin, ICAM-1, VCAM-1, TNF-α, and IL-1β were reduced upon brain death. In order to investigate whether therapeutically targeting C3 would diminish DBD-induced cardiac damage as well, mice were treated with CR2-Crry after receiving a living or DBD heart. CR2-Crry is a complement inhibitor, that targets C3 split products by binding local C3b deposits. Upon treatment with CR2-Crry, recipients who received DBD donor hearts showed reduced cardiac troponin I levels and histological injury scores, similar to levels of living transplanted hearts. Thereby, CR2-Crry treatment diminished neutrophil and macrophage infiltration and prolonged allograft survival of treated DBD donor hearts compared to untreated controls (81).

Based on these studies, it is suggested that complement inhibitory strategies applied to the deceased donor may provide protection of the heart graft. To see whether the results seen in rodent models are clinically relevant, Atkinson et al. analyzed complement deposition in human DBD heart biopsies and living donors. The human biopsies taken from DBD donors before implantation showed C3d complement deposition and inflammation in all grafts, compared to minimal C3d deposition in biopsies from living donor hearts. The complement staining patterns in human DBD hearts demonstrate that complement activation already occurs in the DBD heart, independently from the ischemia-reperfusion phase (80). However, the contribution of each activation pathway of the complement system in DBD heart injury is not fully known. The CP might be involved, since IgM complexes show similar distribution patterns as seen for C3d staining in murine DBD hearts (81). In human heart biopsies from both DBD and living donors stained for C4d, 50% of the cases showed C4d deposition in DBD biopsies before implantation into the recipient. In contrast, living donor hearts showed no C4d deposition at all, which suggests a potential role for the CP in DBD-induced heart injury (80). Whether the other complement activation pathways are involved as well-needs to be further elucidated. Furthermore, the role of the complement system in DCD heart donation requires additional attention. Zhang et al. investigated the role of natural immunoglobulins in a model for myocardial warm IRI and revealed that pre-existing IgM's, which recognize “ischemic antigens,” are the main initiator of pathology through activation of the complement system (82). Regarding variable warm-ischemia times in DCD donors, this might be an interesting field of future research.

## Lung Transplantation

### Challenges in Lung Transplantation

In 2017, 1.233 lung transplants have been performed in Europe, as registered by the Eurotransplant International Foundation (Figure 2). The most important indications for bilateral lung transplantation (LuTx) are Chronic Obstructive Pulmonary Disease (COPD), Cystic Fibrosis (CF), Interstitial Pulmonary Fibrosis (IPF), and Primary Pulmonary Arterial Hypertension (PPAH) (83). Although the number of LuTx is much lower than the numbers of abdominal organs transplanted, donor shortage is an important issue in LuTx as well. This observation is mainly the result of a lower utilization rate of lungs than of abdominal organs. More than 70% of the donor livers and kidneys are procured and used for transplantation, while lungs are only suitable for transplantation in around 20% of the cases (84). Lung injury is an important reason for excluding donor lungs for transplantation and is caused by the process of donor death and complications at the intensive-care unit (85).

Most donor lungs are procured from DBD donors, in which the process of brain death leads to inflammation and pericapillary leakage, resulting in pulmonary edema (86). Those mechanisms of injury make the lung more susceptible to IRI and lead to poor graft survival rates of only 54% after 5 years (87). The last years attempts have been made to enlarge the donor pool. Examples are transplantation of lungs from DCD 3 donors (88), usage of ECD (89), living-donor lobar lung transplantation (90), and application of the technique of *ex vivo* lung perfusion (EVLP) in an attempt to test and repair discarded donor lungs (91, 92). So far, those attempts have demonstrated similar outcomes on graft survival compared to standard DBD lungs (93, 94). However, those efforts have not yet closed the gap between supply and demand in LuTx and did not lead to improved graft survival. In an immunological point of view the lung is an interesting organ, because of continuous exposition to the outside environment, serving as a first line barrier to infection. As a result, the immunomodulation regimen in lung transplant recipients makes those patients more prone to fungal infections (95). This issue emphasizes the challenge to create a balance between infection and rejection. In order to do so, the exact immunological pathways involved in donor lung injury need to be further elucidated before development of novel immunosuppressive strategies, which tackle the issues of donor shortage and graft survival in the field of LuTx.

### The Complement System in Lung Transplantation

Complement in LuTx has mostly been studied in recipients, focused on the role of the complement system on lung IRI and rejection. However, Budding et al. demonstrated that the complement system is already involved from the first step of the lung transplantation process. The study showed that recipients who received a donor lung with a CD59 protein single nucleotide polymorphism (SNP) configuration, had a higher risk for chronic rejection after LuTx. Under normal circumstances CD59 acts as a regulatory protein, which suppresses MAC formation by binding C9 to C5b-C8 complexes, which results into inhibited cell lysis. The presence of a CD59 SNP configuration affects CD59 expression and sensitivity to complement-mediated cell lysis, which increases the risk for rejection in recipients who receive a CD59 SNP donor lung (96). These results suggest that C5b-9 is involved in donor-related lung injury. However, considering the direct effects of complement split-products like C3a and C5a produced in the earlier steps of the complement system, the question is raised whether regulatory proteins of the terminal pathway should be the target of interest. In lungs, C5a, but also C3a, have shown to induce acute pulmonary injury by constriction of smooth muscle walls in bronchioles and pulmonary arteries, and cause focal atelectasis (97). C3a levels in plasma have additionally been described to be associated with later development of acute respiratory distress syndrome (ARDS) in polytrauma patients (98). Furthermore, C3a and C5a are described to attract and activate neutrophils (99, 100). Of importance, since the amount of recruited and infiltrated neutrophils are associated with graft survival after LuTx (101). The beneficial effect of targeting the donor lung on the level of C3a has been demonstrated by Cheng et al. in a mouse model for LuTx. First, the study confirmed that the process of brain death induces donor lung injury. Pathology lung injury scores examining congestion, hemorrhage and inflammation, were significantly increased. Secondly, the amount of infiltrated neutrophils and macrophages were elevated in DBD mice compared to both sham-operated mice and living donor mice. Furthermore, a significantly elevated expression of complement receptor C3a (C3aR) in DBD donor lungs was found. The C3aR was mainly expressed on bronchial and epithelial cells and lung endothelium. Thereafter, targeting the C3aR with a nebulized complement C3a receptor antagonist was tested in a mouse model of LuTx. Recipients of untreated DBD donor lungs showed aggravated IRI and acute rejection (AR) grades, which was ameliorated after treatment with the C3aR antagonist. IRI and AR grades were even returned to levels as seen after living-donor LuTx (102).

Despite the limited amount of research performed on the topic of DBD-related lung injury, it is suggested that the complement system is already involved from the first step of the process, namely the donor. Of particular interest, given the observation that lungs locally produce complement proteins. Pulmonary alveolar type II epithelial cells generate proteins of the CP and AP, in particular C2, C3, C4, C5, and factor B (103). Besides that, human bronchiolar epithelial cells are able to synthesize C3 (104). However, literature on the mechanisms of the complement system in the lung donor and the contribution of local complement production in this pathophysiology is scarce. Which specific pathway should be the target of interest, and if treatment focused on those components will lead to improved graft survival without compromised defense mechanisms against pathogens, needs to be further investigated.

## Future Perspectives

The complement system regained new interest in the field of transplantation and the amount of acquired knowledge is increasing. The complement system was first studied in RTx, revealing its potential role in reperfusion injury and survival. However, new studies elucidated that the complement system already plays a role from the first step of the transplantation process, in the donor (33). Other studies showed that this damage accumulates throughout the rest of the transplantation process (81). Striving to restore an immunologically active organ before implementation in the recipient may have advantages, in favor of transplantation outcomes in patients. The complement system might be a potential therapeutic target for this purpose, and not only in the field of RTx. However, multiple knowledge gaps exist with regards to the role of the complement system throughout the transplantation process. Those need to be elucidated before therapeutics can be implemented in the clinical setting. For some organs, the pace of research developments can be complicated by the small number of transplants performed. The intestines, for example, has only been transplanted 6 times in 2017, as registered by the Eurotransplant International Foundation (Figure 2).

One of the questions that remains unanswered regarding complement-targeted interventions is the optimal timing of drug delivery to the donor graft. Different timing possibilities are (1) treatment in the organ donor, (2) during preservation, or (3) after implementation in the recipient. The main benefit of treating the organ donor is the opportunity to target the immunologically active state of the organs, nearly directly after the damage has occurred. However, it should be considered that all organs will be subjected to the same type and dose of treatment. An important disadvantage of this strategy is the risk that not all donor organs benefit from the same treatment. One organ might benefit from certain therapy, while the other organ might be even negatively affected. This is of particular importance for the liver, given the fine balance between injury and regeneration in this organ (63, 64).

The preservation state, however, provides a window of opportunity to treat the organ in an isolated manner. Various approaches are herein possible, which depends on the method of preservation. Cold static storage is the preservation technique with the longest history and is mostly used (1). Therapeutics can be added to the storage solution or organs can be infused just before cold static storage. The latter approach was demonstrated by Durigutto et al. who infused renal allografts with a targeted complement inhibitor, with beneficial results after reperfusion (49). However, the technique of cold storage is increasingly taken over by *ex vivo* perfusion systems. Various strategies in *ex vivo* perfusion are being practiced such as different perfusion solutions and perfusion temperatures (1). Nevertheless, in all *ex vivo* perfusion strategies blood flow through the organ is mimicked, thereby reducing ischemia times. Besides, oxygen and other additives can be supplemented to the perfusate solution in order to preserve or improve the quality of the organ, which provides opportunities for complement therapeutics as well. In the lungs, even *ex vivo* inhalation of therapeutics can be considered as route of administration. An important benefit of treating the organ in an isolated manner is that a lower treatment dose might be required, especially in in organs with little metabolic activity. This might lower costs of complement-targeted therapies. However, little is known about the approach of treatment during preservation, especially with regards to the effect of treatment on donor-related injury. The few experimental studies that have been performed have mainly focused on preventing or diminishing IRI, by treating unharmed, “healthy” organs with complement inhibitors during the preservation phase (48, 56, 105). Therefore, the question whether inhibiting complement during the preservation phase has an effect on deceased donor-related injury as well, should still be investigated. Furthermore, it should be considered that the complement system might be activated by interaction with foreign materials (106).

Finally, treatment of the organ after implementation in the recipient should be considered as a possible timing of drug delivery. It should be emphasized that donor-related graft injury is amplified by the inevitable event of IRI (81). Beneficial effects of treatment applied in the organ donor or the isolated graft might not cover this second-hit of injury, occurring in the latter phase of the transplantation process. Application of complement-therapeutics in the recipient might tackle this issue, as demonstrated by Ferraresso et al. in a rat model for HTx. They showed that treatment of the recipient with anti-C5 therapy prior to reperfusion, prevented IRI-induced graft injury (107). Nevertheless, possible adverse side effects of complement therapeutics in the recipient need to be elucidated before implementation in the clinics. Finally, it can be considered to combine multiple time points of drug delivery, of which the net effects need to be further studied.

Another unanswered question is the translatability of results from one organ, to other organ systems. Especially since most knowledge on the involvement of complement is gained from research in deceased donor kidneys, it is questioned whether those findings apply to other organs as well. In order to answer this, more studies are needed that focus on unraveling the mechanisms of complement activation, specifically per organ and donor type. Especially, given the physiological dissimilarities between organs and the differences in mechanisms of pathophysiology between DBD and DCD donors. The role of the complement system has been underexposed mostly in DCD donation. Yet this will be of growing importance, since DCD donors are increasingly deployed in an attempt to expand the donor pool.

## Conclusion

In conclusion, it has become evident that the complement system plays an important role in the donor, affecting all potential donor organs. To answer the question whether complement therapeutics should be clinically applied in the multi-organ donor, several uncertainties need to be elucidated first. These uncertainties include the timing, route of drug delivery and optimal target of complement therapeutics, complicated by dissimilarities in the pathophysiology of organs and differences between donor types. A tailor-made approach for each donor organ is pursued, aiming to improve the quality of donor grafts that possibly leads to an enlargement of the donor pool and improved outcomes after transplantation.

## Author Contributions

JvZ and NJ contributed to the conception and design of the work, drafting the work, and agree to be accountable for all the aspects of the work in ensuring that questions related to the accuracy or integrity of any part of the work are appropriately investigated and resolved. MD, ME, HL, and MS revised the work critically for important intellectual content and provide approval for publication of the content.

### Conflict of Interest Statement

The authors declare that the research was conducted in the absence of any commercial or financial relationships that could be construed as a potential conflict of interest.

## Abbreviations

ALAT: Alanine aminotransferase
AP: Alternative pathway
AR: Acute rejection
ARDS: Acute respiratory distress syndrome
ASAT: Aspartate aminotransferase
C1-INH: C1-inhibitor
C3aR: C3a receptor
C5aR1: C5a receptor 1
C5aR2: C5a receptor 2
CF: Cystic fibrosis
CP: Classical pathway
COPD: Chronic obstructive pulmonary disease
CRIg: Complement receptor immunoglobulins
DAMPs: Damage-associated molecular pattern molecules
DBD: Donation after brain death
DCD: Donation after circulatory death
DGF: Delayed graft function
ECD: Extended-criteria donor
HTx: Heart transplantation
IgM: Immunoglobulin
ITx: Intestinal transplantation
IPF: Idiopathic pulmonary fibrosis
IRI: Ischemia-reperfusion injury
LiTx: Liver transplantation
LP: Lectin pathway
LPS: Lipopolysaccharides
LuTx: Lung transplantation
LVAD: Left ventricular assist device
MAC: Membrane attack complex
MASP: Mannan-binding lectin-associated serine protease
MBL: Mannose-binding lectin
RTx: Renal transplantation
PAMPs: Pathogen-associated molecular pattern molecules
PPAH: Primary pulmonary arterial hypertension
sC5b-9: Soluble C5b-9
SCD: Standard-criteria donor
sCR1: Soluble complement receptor-1
SNP: Single nucleotide polymorphism
UW: University of Wisconsin.
